# Correction: Lin et al. Prospects and Challenges of Lung Cancer Vaccines. *Vaccines* 2025, *13*, 836

**DOI:** 10.3390/vaccines14080675

**Published:** 2026-08-04

**Authors:** Zhen Lin, Zegang Chen, Lijiao Pei, Yueyun Chen, Zhenyu Ding

**Affiliations:** 1 Department of Biotherapy, Cancer Center and State Key Laboratory of Biotherapy, West China Hospital, Sichuan University, Chengdu 610041, China; linzhenzlk@wchscu.edu.cn (Z.L.); peilijiao1701@wchscu.edu.cn (L.P.); chenyueyun@wchscu.edu.cn (Y.C.); 2 Department of Oncology, Chongqing Liangping District People’s Hospital, Liangping, Chongqing 405200, China; chenzegangd@163.com

The authors would like to make the following corrections to this published paper [1].

In the original publication, there was a mistake in Reference 136 as published. Reference 136 cited the preprint version of the article. The title of the preprint and the arXiv identifier were correctly provided; however, the author information was inaccurate. The cited work has now been formally published and indexed in PubMed.

Therefore, the original Reference 136, “Wang, J.; Yang, Y.; Wu, J.; Zhao, H.; Xu, Y. Advancements in programmable lipid nanoparticles: Exploring the four-domain model for targeted drug delivery. arXiv 2023, arXiv:2408.05695”, should be replaced with the following corrected reference: “Liu, Z.; Chen, J.; Xu, M.; Ho, S.; Wei, Y.; Ho, H.-P.; Yong, K.-T. Engineered multi-domain lipid nanoparticles for targeted delivery. *Chem. Soc. Rev.* **2025**, *54*, 5961–5994. https://doi.org/10.1039/D4CS00891J. PMID: 40390667.”

In addition, there was a mistake in Table 1 as published. In the peptide vaccine section of Table 1, the fourth entry and the last entry both referred to the same clinical trial, NCT06751901. This duplicate entry may have occurred during the manual compilation and formatting of the table. The last duplicate entry for NCT06751901 should be removed, and the fourth entry for NCT06751901 should be retained.

The corrected Table 1 appears below.

**Table 1:** Summary of ongoing clinical studies on lung cancer vaccines.

Grouping	NCT Number/	Population	Line	Interventions	Phases	Enrollment	Primary Outcomes	Secondary Outcomes	Target Antigen
Peptide Vaccine	NCT05950139	Advanced ALK + NSCLC	1	Vaccine	I/II	12	TRAEs/Immune Response	/	/
	NCT03879694	Metastatic Neuroendocrine Tumors (NETs)	2	Vaccine + Octreotide Acetate + Sargramostim	I	14	TRAEs	ORR/PFS/DOR/Immune Response/Rate of Progression	Survivin
	NCT01720836	NSCLC	1	Vaccine + PolyICLC	I/II	30	Immunologic Response	TRAEs/PFS/OS/Anti-MUC1 Immunity	Mucin1
	NCT06751901	Advanced NSCLC	3	Vaccine + ICIs + Radiotherapy	II	10	ORR/DCR/TRAEs	PFS/OS	Personalized Neoantigen
	NCT05254184	Advanced ALK + NSCLC	2	Vaccine + Nivolumab + Ipilimumab + PolyICLC	I	12	TRAEs	PFS/Immune Response	KRAS
	NCT05269381	Advanced Solid Tumors	2	Cyclophosphamide + Neoantigen Peptide Vaccine + Pembrolizumab + Sargramostim	I/II	36	TRAEs	The Percentage of Immunogenicity Responders and Trial Completers	Personalized Neoantigen
	NCT06095934	EGFR + Advanced NSCLC	2	Chemotherapy + Vaccine + PD-1 Monoclonal Antibody	II	20	ORR	PFS/OS	Personalized Neoantigen
	NCT06472245	Metastatic NSCLC	3	Arm A: OSE2101 Arm B: Docetaxel	III	363	OS	/	P53, HER-2, CEA, MAGE-2 and MAGE-3, and One Pan-HLA DR Binding Epitope
	NCT06202066	Metastatic Neuroendocrine Tumors	2	Part 1: Temozolomide + Vaccine Part 2: Arm a: Temozolomide Arm b: Temozolomide + Vaccine	II	132	Part 1: PFS/TRAEs Part 2: PFS	Part 1: ORR/OS/TTP Part 2: TTP/TRAEs/ORR/OS	Survivin
	NCT04266730	Advanced NSCLC or SCCHN	2	Pembrolizumab + Vaccine	I	6	TRAEs	ORR/OS/PFS	Personalized Neoantigen
	NCT05344209	Advanced or Metastatic NSCLC	1	PD-1/PD-L1-Treatment ± UV1 Vaccination	II	138	PFS	TRAEs	hTERT
	NCT04298606	Stage IB-IIIA Lung Cancer	Postoperative	Vaccine	I	60	≥3 Grade TRAEs/Biomarker Analysis	Quality-of-Life Score	EGF
	NCT05104515	Locally Advanced or Metastatic NSCLC, Ovarian Cancer, and Prostate Cancer	≥2	Vaccine	I	36	TRAEs	ORR	Survivin
	NCT06015724	Refractory NSCLC/ Pancreatic Ductal Adenocarcinoma	≥2	Daratumumab + Nivolumab + Vaccine	II	54	ORR	TRAEs/PFS/OS/DOR	KRAS
	NCT06752044	Advanced NSCLC	2	Radiotherapy + Pd-1 + Vaccine	/	10	ORR/DCR/TRAEs	PFS/OS	Personalized Neoantigen
mRNA Vaccine	NCT03908671	Advanced Esophageal Cancer or NSCLC	/	Vaccine	I	24	TRAEs	DCR/PFS/OS	Personalized Neoantigen
	NCT05142189	NSCLC	/	Vaccine Alone or Vaccine + Cemiplimab/Docetaxel/Carboplatin/Paclitaxel/BNT316/Anti-B7-H3 Antibody/Anti-HER3 Antibody	I	220	DLTs/TRAEs	ORR/DOR/PFS/OS	MAGE A3, CLDN6, KK-LC-1, PRAME, MAGE A4, MAGE C
	NCT06928922	Advanced Lung Cancer	≥2	Vaccine or Vaccine + PD-1	I	22	DLTs/MTD/TRAEs	ORR/DOR/PFS/OS	/
	NCT05557591	Advanced NSCLC	1	Arm A: Cemiplimab Arm B: Cemiplimab + vaccine	II	100	ORR	DOR/PFS/OS/TRAEs	MAGE A3, CLDN6, KK-LC-1, PRAME, MAGE A4, MAGE C
DNA Vaccine	NCT04397003	ES-SCLC	1	Durvalumab + Chemotherapy + Vaccine	II	20	TRAEs	ORR/DOR/PFS/OS	Personalized Neoantigen
	NCT05242965	Advanced NSCLC	Maintenance Therapy	Arm I: Vaccine + Sargramostim Arm II: Sargramostim	II	40	CD8+ TIL/TRAEs	Immune Response/ORR/PFS/OS	CD105/Yb-1/SOX2/CDH3/MDM2
DC Vaccine	NCT05886439	Advanced NSCLC or ES-SCLC	2	Vaccine + Pembrolizumab or Durvalumab	I	40	DLT/TRAES	ORR/DOR/PFS/OS	Personalized Neoantigen
	NCT04147078	Postoperative Locally Advanced Gastric Cancer, Hepatocellular Carcinoma, Lung Cancer and Colorectal Cancer	Postoperative	Vaccine	I	80	DFS	OS/TRAEs	Personalized Neoantigen
	NCT06329908	Advanced Lung Cancer	≥2	Vaccine + ICIs	I	20	TRAEs	ORR/PFS	Personalized Neoantigen
	NCT05195619	Advanced or Recurrent Metastatic NSCLC	Stable Disease after Immunotherapy or Targeted Therapy	Vaccine + ICIs or Targeted Therapy	I	16	TRAEs	ORR/DOR/PFS/OS	Personalized Neoantigen
	NCT06752057	Advanced NSCLC	2	Radiotherapy + Pd-1 + Vaccine	/	10	ORR/DCR/TRAEs	PFS/OS	Personalized Neoantigen

Abbreviation: TRAEs: treatment-related adverse events, ORR: overall response rate, PFS: progression-free survival, DOR: duration of response, OS: overall survival, TTP: time to progression, DCR: disease-control rate, DLTs: dose-limiting toxicities, MTD: maximum tolerated dose, KRA: Kirsten rat sarcoma viral oncogene homolog, P53: tumor protein p53, HER-2: human epidermal growth factor receptor 2, HER-3: human epidermal growth factor receptor 3, CEA: carcinoembryonic antigen, MAGE: melanoma antigen gene 2, MAGE A3: melanoma antigen gene A3, MAGE A4: melanoma antigen gene A4, MAGE C: melanoma antigen gene C, hTERT: human telomerase reverse transcriptase, PRAME: preferentially expressed antigen in melanoma, CLDN6: claudin 6, KK-LC-1: Kita-Kyushu lung cancer antigen-1, Pd-1: programmed cell death protein 1, ICIs: immune checkpoint inhibitors, CD8+ TIL: cluster of differentiation 8 tumor-infiltrating lymphocytes, Poly-ICLC: polyinosinic-polycytidylic acid stabilized with polylysine and carboxymethylcellulose, mRNA: messenger ribonucleic acid, DNA: deoxyribonucleic acid, SCCHN: squamous cell carcinoma of head and neck, NSCLC: non-small cell lung cancer, ALK: anaplastic lymphoma kinase, EGFR: epidermal growth factor receptor.

The authors state that the scientific conclusions are unaffected. These corrections were approved by the Academic Editor. The original publication has also been updated.
